# Neural Intrinsic Timescales in the Macaque Dorsal Premotor Cortex Predict the Strength of Spatial Response Coding

**DOI:** 10.1016/j.isci.2018.11.033

**Published:** 2018-11-22

**Authors:** Rossella Cirillo, Valeria Fascianelli, Lorenzo Ferrucci, Aldo Genovesio

**Affiliations:** 1 Department of Physiology and Pharmacology, Sapienza - University of Rome, Piazzale Aldo Moro 5, Rome 00185, Italy; 2 Institut des Sciences Cognitives Marc Jeannerod - UMR 5229, 67 Boulevard Pinel, Bron Cedex 69675, France; 3 PhD Program in Behavioral Neuroscience, Sapienza University of Rome, Rome, Italy

**Keywords:** Cognitive Neuroscience, Neuroscience

## Abstract

Our brain continuously receives information over multiple timescales that are differently processed across areas. In this study, we investigated the intrinsic timescale of neurons in the dorsal premotor cortex (PMd) of two rhesus macaques while performing a non-match-to-goal task. The task rule was to reject the previously chosen target and select the alternative one. We defined the intrinsic timescale as the decay constant of the autocorrelation structure computed during a baseline period of the task. We found that neurons with longer intrinsic timescale tended to maintain a stronger spatial response coding during a delay period. This result suggests that longer intrinsic timescales predict the functional role of PMd neurons in a cognitive task. Our estimate of the intrinsic timescale integrates an existing hierarchical model (Murray et al., 2014), by assigning to PMd a lower position than prefrontal cortex in the hierarchical ordering of the brain areas based on neurons' timescales.

## Highlights

• The spatial response encoding during a delay depends on neurons' timescales
• Longer intrinsic timescales foretell the role of PMd neurons in a cognitive task
• PMd occupies a lower level than PF in the hierarchical organization of brain areas

## Graphical Abstract

**Figure:**
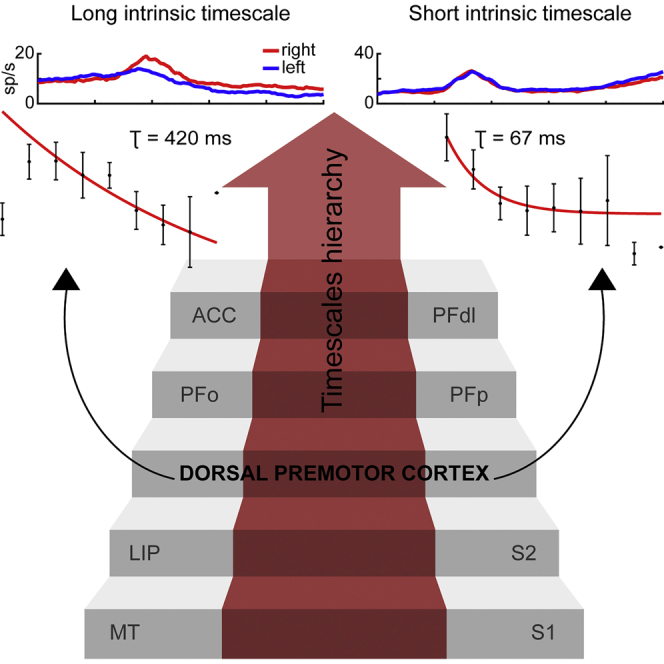

## Introduction

Neurons in different cortical areas are characterized by differences in the temporal stability of their firing rates (Ogawa and Komatsu, 2010; Murray et al., 2014) that represents the ability of a neuron to sustain its firing rate over time, computed as the decay time constant of the autocorrelation structure during a baseline period. We refer to it as “neural intrinsic timescale.” For example, neurons in the frontal eye field (FEF) exhibited higher temporal stability compared with the ones in area V4. This result is consistent with the ability of neurons in FEF (Bruce and Goldberg, 1985, Chafee and Goldman-Rakic, 2000), but not V4 (Bisley et al., 2004), to maintain visual information in time without the presence of a visual stimulus.

Murray et al. (2014), analyzing multiple datasets, went further and found a matching between the hierarchy of brain areas based on their intrinsic timescales and their position in the hierarchy based on anatomical connectivity (Felleman and Van Essen, 1991). In this hierarchy, the prefrontal cortex (PFC) has the longest timescales; the posterior parietal cortex, the intermediate timescales; and the sensory cortex at the bottom of the hierarchy, the shortest timescales. The longest timescale, based on the examination of two datasets, was identified for the median prefrontal anterior cingulate cortex (ACC) (Murray et al., 2014).

Other studies, using another approach, instead of focusing on the heterogeneity of timescales across areas, examined differences in intrinsic timescales across neurons within the same cortical area (Nishida et al., 2014, Cavanagh et al., 2016, Fascianelli et al., 2017), to address whether distinct functional classes of neurons might also show timescale differences based on their specific functions.

In the lateral intraparietal (LIP) area, Nishida et al. (2014) found that neurons with delay activity specialized to maintain information presented slow intrinsic timescales. Similar results were later described in the PFC (Cavanagh et al., 2016, Fascianelli et al., 2017), but with differences between the PF subdivisions between the two studies, which could be explained by task differences. Fascianelli et al. (2017) found a similar specialization of neurons with longer intrinsic timescales for maintaining spatial information in the delay and feedback periods in the dorsolateral prefrontal cortex (PFdl), but not in the orbital prefrontal cortex (PFo) or the polar prefrontal cortex (PFp). On the other side, Cavanagh et al. (2016) reported a similar relationship between longer intrinsic timescales and chosen value neurons in the PFo, but not in the PFdl. One interpretation to account for discrepancies between these studies is that the temporal specialization generated by the intrinsic properties of self-sustained activity is not necessary for all types of computations in the PF, but only for specific functions in each area, namely, spatial working memory and monitoring processes in PFdl and value computations in PFo. Similar to PFC, PMd neurons also show persistent activity (Kurata and Wise, 1988, Mushiake et al., 1991, di Pellegrino and Wise, 1993, Crammond and Kalaska, 2000), integrating different information through time (Hoshi and Tanji, 2000). However, PMd-intrinsic timescales have not been assessed yet. Nevertheless, previous studies (Nakayama et al., 2008, Arimura et al., 2013) have compared the duration of PMd and PFdl activities for behavioral goals and actions. These studies showed comparable signal durations in PMd and PFdl, in contrast to shorter signal durations observed in ventrolateral prefrontal cortex and the globus pallidus. Using our dataset on the non-match-to-goal (NMTG) task (Cirillo et al., 2018), we addressed the study of the intrinsic timescales of single neurons by computing their intrinsic features during the baseline period. Using the baseline period prevents our analysis from being influenced by relevant task parameters, and leading to define them as “intrinsic timescales.” In particular, we focused on the baseline activity during the holding central stimulus epoch at the beginning of each trial. This particular task epoch was suitable for the assessment of the intrinsic ability of PMd neurons to maintain a persistent activity, by preceding any stimulus-related effect on the discharge rate.

We first tested whether the delay activity for the spatial response in PMd depended upon each neuron's intrinsic timescale, as shown in PFdl for the spatial response (Fascianelli et al., 2017), and in PFo for value information (Cavanagh et al., 2016). Second, we computed the PMd timescales for comparison with those of the other brain areas. The neural intrinsic timescale of the PMd was calculated using the NMTG task in a baseline period. Here, we computed the intrinsic timescale of PMd, made a comparison between PMd's and other frontal areas' intrinsic timescales calculated in previous studies, and tested whether longer intrinsic timescales of single PMd neurons were associated with stronger spatial coding properties during the delay period of the task.

## Results

**Figure 1:**
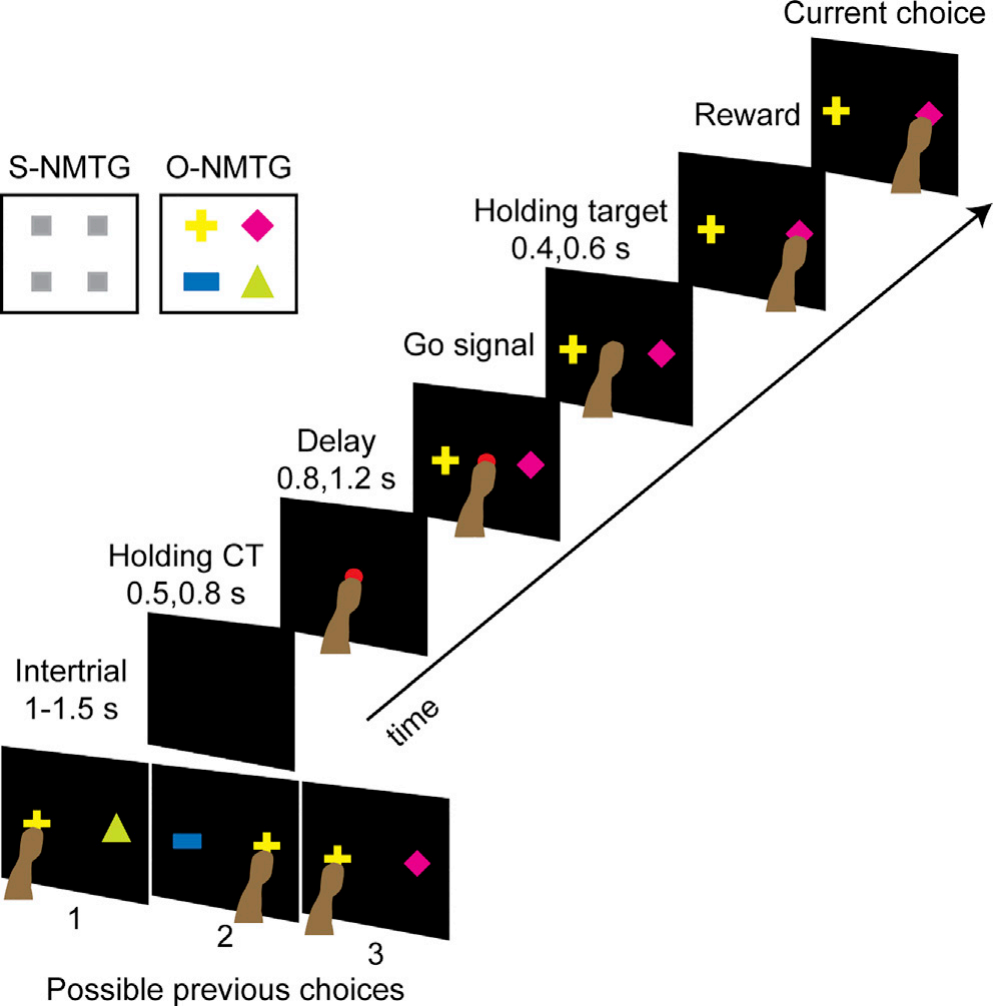
Behavioral Task Temporal sequence of events of one example trial of the object non-match-to-goal task (O-NMTG). Left panel shows the peripheral targets used during the spatial and object versions of the task. In both cases, the correct choice depended on the previous choice. Three possible previous condition combinations and choices (1, 2, and 3) appear on the bottom part of the figure.

Figure 1 illustrates the spatial NMTG (S-NMTG) and object NMTG (O-NMTG) tasks described in a previous work (Cirillo et al., 2018) and in more details in Methods.

Briefly, these two tasks differed only in the peripheral stimuli: in the S-NMTG two peripheral targets, i.e., two identical gray rectangles, appeared in two of four possible screen positions, whereas in the O-NMTG the peripheral targets were four objects (differing in color and shape) that appeared randomly paired one to the left and one to the right of the central stimulus. In both versions, the task rule was to reject the previously selected target and choose the alternative one. Both monkeys performed the NMTG task accurately. Performance was 91% ± 1% for monkey 1 and 90% ± 1% (±SEM) for monkey 2. For the analysis on the S-NMTG task, we decided to collapse the center and bottom positions of the same side of the screen (with respect to the central stimulus), and they were assigned either to the right or to the left position to make the analysis of this task version comparable with the O-NMTG task, which included only two target positions (right and left).

The database for this study consisted of 328 neurons (210 and 118 from monkey 1 and monkey 2, respectively) that were selected by using the single-unit stability method described in Cirillo et al. (2018).

### Neural Intrinsic Timescale Assigned to Neural Population and Single Neurons

**Figure 2:**
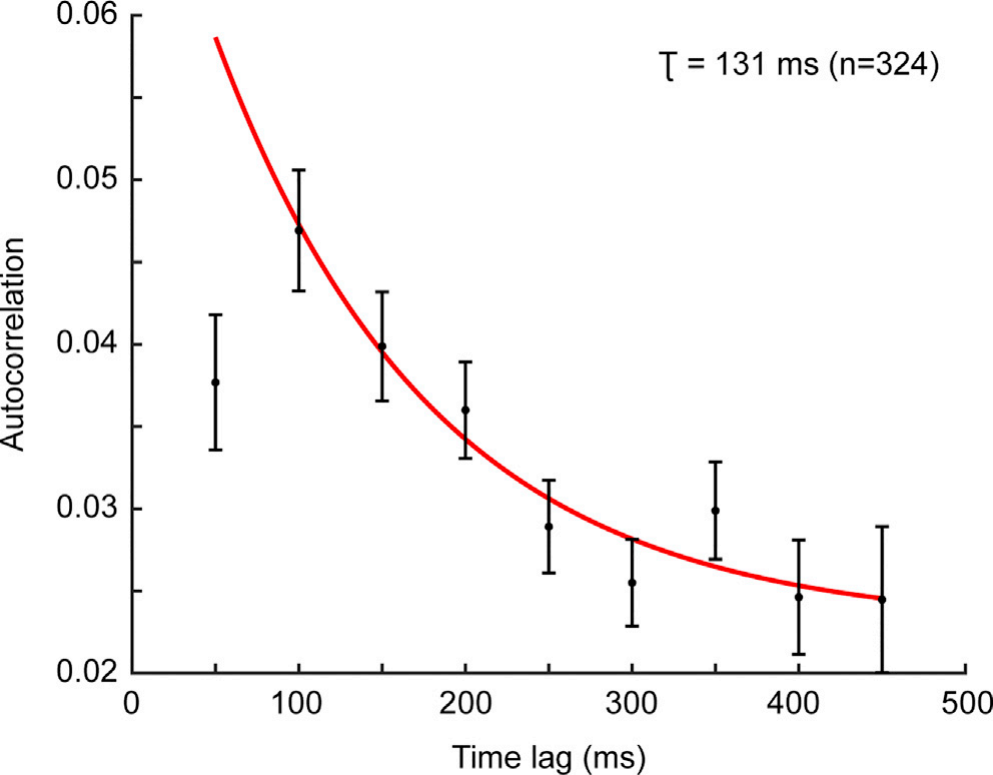
Autocorrelation Structure Mean spike count autocorrelation values computed using 50-ms time bins in a 500-ms time window of the baseline as a function of time lags (mean ± SEM). The solid red line represents the exponential fit. The autocorrelation value at the shortest time lag of 50 ms shows refractory adaptation, and it has been excluded from the fit procedure. The intrinsic timescale obtained from the exponential fit is shown on the top right corner.

We assessed the intrinsic timescale *τ* of the neural population by estimating the decay time constant of the autocorrelation structure during the baseline period. In particular, 324/328 neurons satisfied the requirements listed in the Methods. Figure 2 shows the autocorrelation structure of PMd (averaged across neurons and time lags) during the baseline period at different time lags. We superimposed the exponential fit that gave us an estimate of the intrinsic timescale *τ* = 131 ms (14, 247 ms) at 95% confidence level.

To study the relationship between the intrinsic timescale and the strength of neuronal selectivity, we calculated the intrinsic timescale for each neuron during the baseline period and their area under receiver operating characteristic curve (auROC) for the spatial response (right-left) during the delay period.

**Figure 3:**
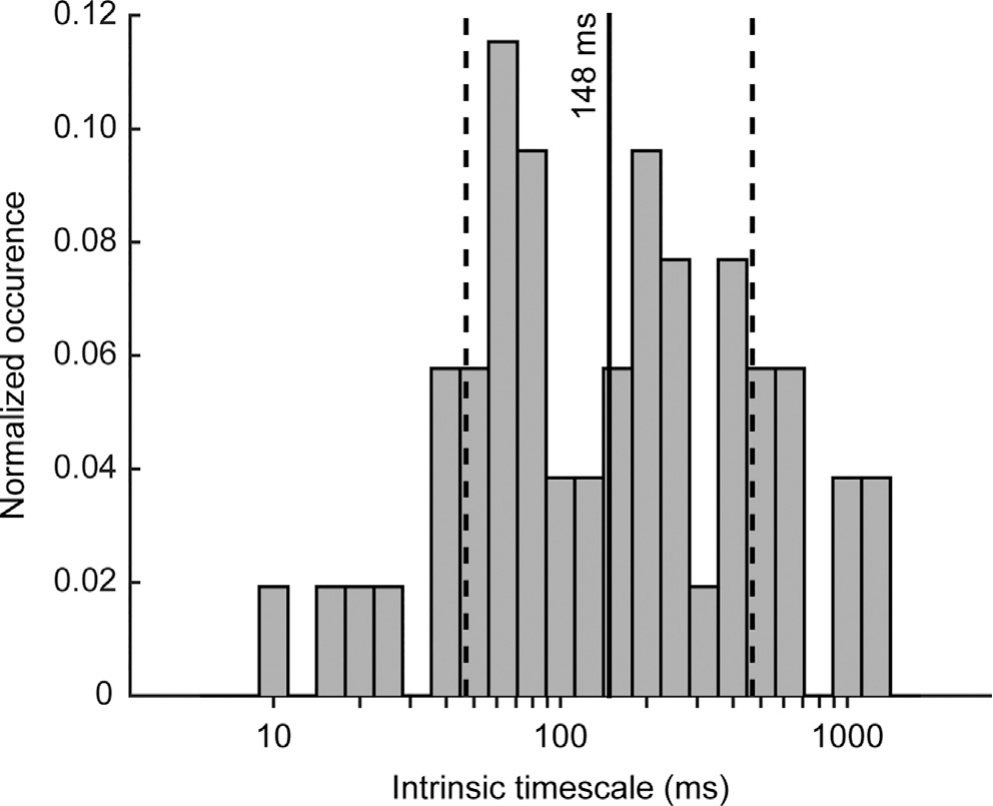
Intrinsic Timescale Distribution A high heterogeneity was present within the neural population (n = 52). Solid and dashed vertical lines are the mean (log(τ)) and mean (log(τ)) ± SD (log(τ)), respectively. The intrinsic timescale distribution was computed in the baseline period.

To assess the intrinsic timescale for each neuron, after the fitting procedure, we removed neurons with a negative intrinsic timescale or a negative amplitude (see Methods). Specifically, 314/324 neurons met these requirements. We further selected the remaining neurons by requiring an adjusted R^2^, and we ended up with 60 neurons with a mean of the adjusted R^2^ of 0.8 ± 0.1 (mean ± SD). From this population of 60 neurons, we further removed the outliers according to the *τ* distribution using the interquartile method (see Methods). We finally selected a neural sample of 52 neurons. We found a high heterogeneity in the distribution of the intrinsic timescale of each neuron (Figure 3). We found a mean value of the intrinsic timescale of 148 ± 3 ms (mean ± SD), compatible with the timescale value we estimated at the population level (131 ms, Figure 2).

### Multiple Linear Regression on Neural Population

We performed a multiple linear regression analysis on the population of 324 neurons to test the relationship between the mean firing rate and the autocorrelation values computed in the 500-ms baseline window and the strength of neuronal selectivity for the spatial response calculated in the delay period (see Methods).

We regressed the autocorrelation values at three time lags and the mean firing rate onto the auROC of each neuron and observed that the contribution of the autocorrelation value to the neuronal selectivity was 100 times stronger than the firing rate weight at 100- and 200-ms time lags (see Table 1). Only for the 300-ms time lag, the coefficient of the autocorrelation value was not significantly different from 0 (p = 0.09; Table 1). These results indicate that neurons sustained their activity within time lags smaller than 200 ms, in accordance with the value below 200 ms of the intrinsic timescale estimated for the neural population (Figure 2).

**Table 1:** Regression Analysis

Time Lag (ms)	Coefficient	p Value
100	FR = 0.002	0.02
	ρ = 0.14	0.02
200	FR = 0.002	0.02
	ρ = 0.21	0.003
300	FR = 0.002	0.008
	ρ = 0.15	0.09

Results of the multiple linear regression analysis on 324 total neurons. The autocorrelation values (*ρ*) computed at three time lags and the mean firing rates (FR) computed during the baseline period were regressed onto the auROC of each neuron.

### Relationship between the Neural Intrinsic Timescale and the Strength of Neural Selectivity

**Figure 4:**
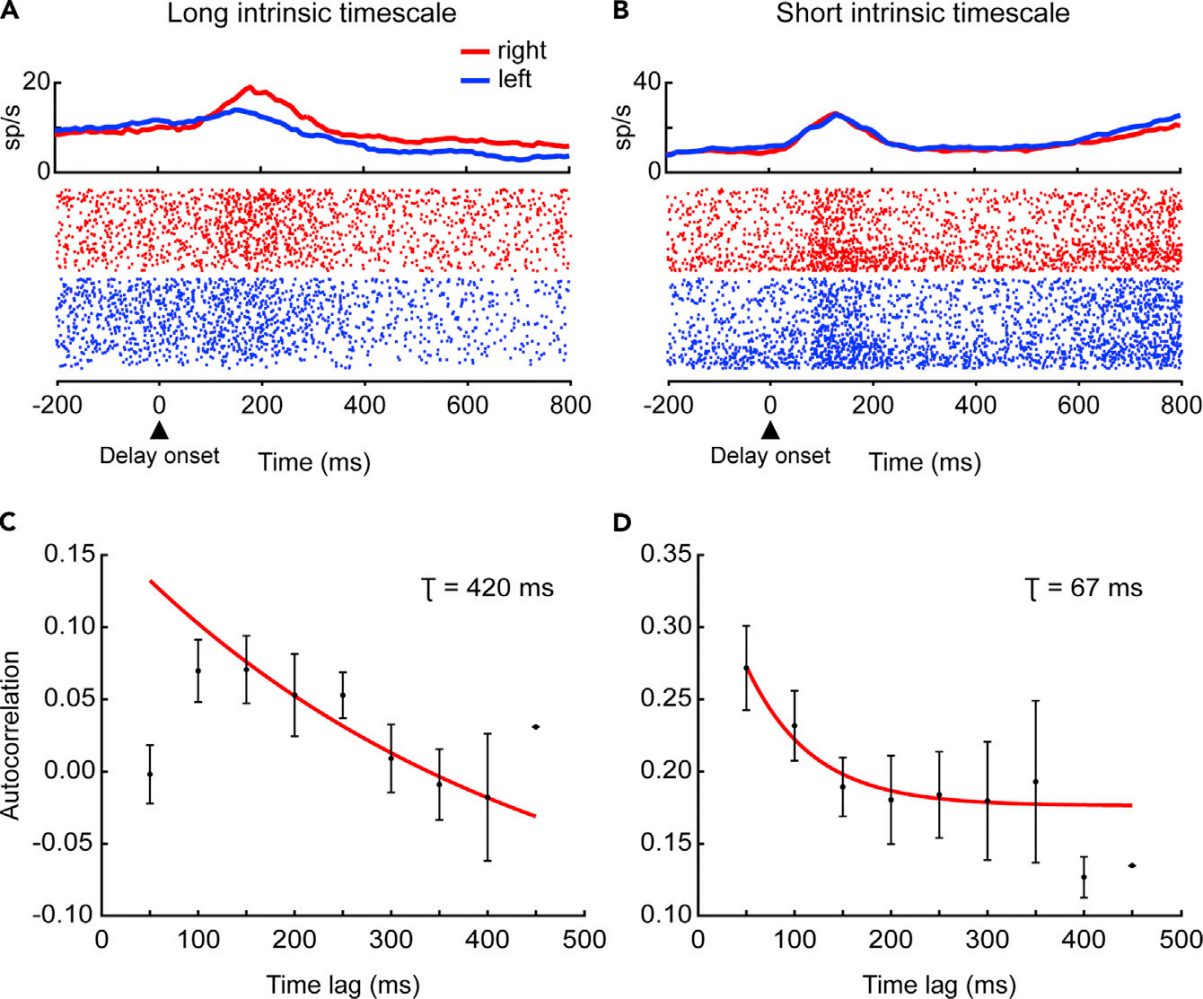
Example Neurons Example of two neurons with long and short intrinsic timescales. (A–C) Example neuron with long intrinsic timescale (*τ* = 420 ms) and response selectivity in the delay period. (A) Raster plot for right (red) and left (blue) trials aligned to the delay onset. Each dot indicates the occurrence of a spike. The average firing rates shown on top of the raster plot were computed using a 100-ms time window size stepped by 10 ms. (C) Mean autocorrelation values (mean ± SEM) as a function of time lag computed in the baseline period. The red line is the exponential fit. (B–D) Example neuron with short intrinsic timescale (*τ* = 67 ms) and not response selective in the delay period. (B) The same as in A. (D) The same as in C.

To assess the strength of neural selectivity, we computed the normalized auROC for the spatial response in the time window 400–800 ms from the peripheral targets' onset. We first tested, as a control, the relationship between both the intrinsic timescale and the mean firing rate computed in the baseline period and the auROC. The neurons' intrinsic timescales and the mean firing rates were regressed onto the auROC of each neuron for the population of 52 neurons to which the intrinsic timescale was assigned. We found a significant relation between the intrinsic timescales and the auROC values (p = 0.009), but not between the firing rate and auROC (p > 0.05). To further examine the relationship between the individual intrinsic timescale and the strength of the neural selectivity, we divided the 52 neurons into two groups based on the magnitude of their intrinsic timescales (see Methods). Figure 4 shows two PMd neurons with long and short intrinsic timescales, respectively. Only the neuron with long intrinsic timescale (*τ*) computed during the baseline period (τ = 420 ms, Figure 4C) encoded the spatial response with higher activity for the right choice during the delay period (Figure 4A). Conversely, the example neuron with the short intrinsic timescale (*τ* = 67 ms, Figure 4D) was not spatial selective (Figure 4B).

**Figure 5:**
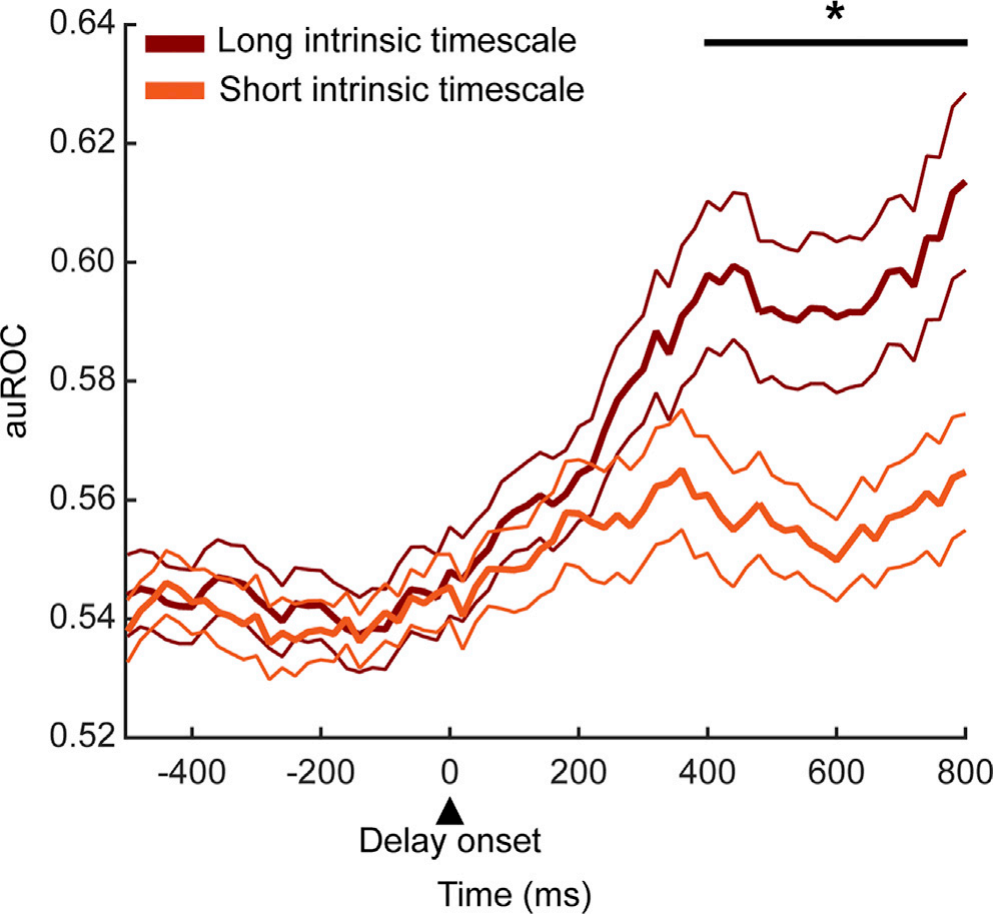
Population auROC Values Time course of the auROC for the spatial response aligned on the peripheral targets' onset. The population of neurons with long intrinsic timescale (dark red curve, mean ± SEM, n = 26) shows higher auROC values for the spatial response than those with short intrinsic timescale (orange curve, mean ± SEM, n = 26). The black line indicates a significant difference in the 400- to 800-ms time window of the delay period (Kruskal-Wallis, p = 0.04).

Next, we compared at the population level the temporal dynamics of the auROC values of the two populations with short and long intrinsic timescales (see Methods). Based on multiple linear regression results, we expected that the latter neuronal population would show stronger spatial response selectivity than the former one. Figure 5 shows the time course of the response selectivity during the delay period. The population with longer intrinsic timescales (dark red curve, mean ± SEM, n = 26) showed stronger response selectivity than the population with shorter intrinsic timescales (orange curve, mean ± SEM, n = 26) in the 400- to 800-ms time window of the delay period (Kruskal-Wallis test, p = 0.04). We next investigated the relationship between the intrinsic timescales of the 52 total neurons and their auROC values (Figure S1). We found a moderate but significant correlation between these two variables (Pearson correlation coefficient: r = 0.30; p = 0.03), which means that, at the population level, auROC values are weakly affected by intrinsic timescales. As a further control, we repeated this analysis after discarding 7 neurons of the 52 total neurons that were selective for the response of the previous trial. We confirmed the result for this smaller population (n = 45; Kruskal-Wallis test, p = 0.03).

## Discussion

This study examined the relationship between intrinsic timescales of PMd neurons in a baseline period of an NMTG task and the strength of neural coding of spatial responses during the delay. It is still debated whether the mechanisms that allow to maintain a persistent activity can depend more on the intrinsic biophysical properties of a neuron or on their connectivity (for a review, see Zylberberg and Strowbridge, 2017). Here, we found that the intrinsic neural specialization for temporally extended computations expressed by the neural intrinsic timescales in PMd predicted the neuron's involvement in maintaining spatial information: neurons with longer intrinsic timescales tended to show a stronger spatial response modulation in the delay period.

The existence of neurons with different timescales responds to the need to gather and integrate information over multiple timescales. In a fast changing environment it could be appropriate to track changes at short timescales, whereas in more stable environments it would be better to rely on long timescales. For example, using functional magnetic resonance imaging in humans (Hasson et al., 2008, Lerner et al., 2011), it has been shown that brain areas differently accumulate information over time, with higher-order areas being more influenced by prior sensory information than lower-order areas in a hierarchy of information processing. In monkeys, neurons can hold information with a range of different timescales as described and quantified in different cortical areas (Bernacchia et al., 2011, Arimura et al., 2013), which include PMd. What is striking is that differences in intrinsic timescales between brain areas can also be predictive of their coding timescales. Considering comparisons between brain areas, Murray et al. (2017) examined datasets from the LIP area, the ACC, and the PFC and found that these brain areas showed a ranking of reward-coding timescales that was in line with the ranking of their intrinsic timescales.

Therefore, neurons in different brain areas not only differ for the duration of their coding activity but also for their intrinsic timescales.

In this study, we focused our analysis on the intrinsic timescales calculated during baseline but by looking at the specialization of neurons within the same area instead of focusing on differences between brain areas. We hypothesized that neurons exhibiting a long intrinsic timescale would carry more information during a delay period. Indeed, intrinsic timescales can vary between neurons even within the same brain area depending on their functional properties. Importantly, it has been shown (Ogawa and Komatsu, 2010, Murray et al., 2014) that functional hierarchies can be determined not only based on the laminar pattern of long-term projections (Felleman and Van Essen, 1991, Barbas and Rempel-Clower, 1997) but also by taking into account the intrinsic timescales of neural activity. The importance of timescales for generating functional hierarchies has been shown by the neural network model of Yamashita and Tani (2008) in which functional hierarchies emerged not only because of the spatial connectivity between neurons but also by introducing units with slow and fast timescales.

Our results show that longer intrinsic timescales in PMd were predictive of stronger neural selectivity for the spatial response, similarly to what was found using a strategy task (Tsujimoto et al., 2011, Tsujimoto et al., 2012, Tsujimoto and Genovesio, 2017, Fascianelli et al., 2017).

We also measured the intrinsic timescale of the PMd neuronal population for comparison with other cortical areas, adding PMd to the group of cortical areas previously examined to quantify their intrinsic timescales (Murray et al., 2014, Fascianelli et al., 2017). Our results show that the intrinsic timescale of PMd was shorter than the timescales of prefrontal areas (>150 ms on average), with the exception of the timescale calculated for PFdl on the Pasternak's dataset (Murray et al., 2014), but longer than the timescale of primary somatosensory cortex (S1; ∼50 ms) and medial-temporal area in visual cortex (∼70 ms). This result expands the hierarchy of intrinsic timescales of the cortical areas sketched by Murray et al. (2014) and extended by Fascianelli et al. (2017) to PFdl, PFp, and PFo, thus assigning to PMd a slightly lower hierarchical level than PFC areas. However, a modeling study by Chaudhuri et al. (2015) suggests caution to assign definitive hierarchies based on timescales. By contrasting the responses of the model to visual versus somatosensory stimulation, they found that different timescales could emerge based on input modality. Therefore, future studies will have to define neurons' timescales consistency through tasks, preferably in the same study, to evaluate whether the functional hierarchy between areas based upon their intrinsic timescales might vary depending on the type of information processed.

In conclusion, we showed that the relevance of long intrinsic timescales of PMd neurons could be that of holding spatial response information over time. Our results also integrate the hierarchical model of intrinsic timescales proposed by Murray et al. (2014), assigning to PMd a lower hierarchical level than PFC areas. This result seems in conflict with the hierarchy between source and target areas, based on the fraction of projections originating from the supragranular layers of the source area (the supragranular layer neurons, SLN), and whereby PMd appears on top of PFC (Murray et al., 2014). This mismatch should be further evaluated by examining the timescales of other brain areas to assess consistency between hierarchies.

### Limitations of the Study

We analyzed the temporal stability of PMd neurons looking at the autocorrelation structure of the spiking activity during the baseline period. The first limitation, common to studies on timescales, concerns the difficulty to rule out, even in a baseline epoch, the influence on the calculation of the timescales of all the possible task-related neural modulations, e.g., the elapsing time from the beginning of the trial or the temporal expectation of the cue signal. The second limitation is that it remains unclear whether the peculiarity of the NMTG task and its complexity contributed to the results obtained, or if another spatial cognitive task could have led to the same conclusions.

## Methods

All methods can be found in the accompanying Transparent Methods supplemental file.

## Supplemental Information

Document S1. Transparent Methods and Figure S1

## References

[bib1] Arimura N., Nakayama Y., Yamagata T., Tanji J., Hoshi E. (2013). Involvement of the globus pallidus in behavioral goal determination and action specification. J. Neurosci..

[bib2] Barbas H., Rempel-Clower N. (1997). Cortical structure predicts the pattern of corticocortical connections. Cereb. Cortex.

[bib3] Bernacchia A., Seo H., Lee D., Wang X.J. (2011). A reservoir of time constants for memory traces in cortical neurons. Nat. Neurosci..

[bib4] Bisley J.W., Zaksas D., Droll J.A., Pasternak T. (2004). Activity of neurons in cortical area MT during a memory for motion task. J. Neurophysiol..

[bib5] Bruce C.J., Goldberg M.E. (1985). Primate frontal eye fields. I. Single neurons discharging before saccades. J. Neurophysiol..

[bib6] Cavanagh S.E., Wallis J.D., Kennerley S.W., Hunt L.T. (2016). Autocorrelation structure at rest predicts value correlates of single neurons during reward-guided choice. Elife.

[bib7] Chafee M.V., Goldman-Rakic P.S. (2000). Inactivation of parietal and prefrontal cortex reveals interdependence of neural activity during memory-guided saccades. J. Neurophysiol..

[bib8] Chaudhuri R., Knoblauch K., Gariel M.-A., Kennedy H., Wang X.-J. (2015). A large-scale circuit mechanism for hierarchical dynamical processing in the primate cortex. Neuron.

[bib9] Cirillo R., Ferrucci L., Marcos E., Ferraina S., Genovesio A. (2018). Coding of self and other’s future choices in dorsal premotor cortex during social interaction. Cell Rep..

[bib10] Crammond D.J., Kalaska J.F. (2000). Prior information in motor and premotor cortex: activity during the delay period and effect on pre-movement activity. J. Neurophysiol..

[bib12] di Pellegrino G., Wise S.P. (1993). Visuospatial versus visuomotor activity in the premotor and prefrontal cortex of a primate. J. Neurosci..

[bib13] Fascianelli V., Tsujimoto S., Marcos E., Genovesio A. (2017). Autocorrelation structure in the macaque dorsolateral, but not orbital or polar, prefrontal cortex predicts response-coding strength in a visually cued strategy task. Cereb. Cortex.

[bib14] Felleman D.J., Van Essen D.C. (1991). Distributed hierarchical processing in the primate cerebral cortex. Cereb. Cortex.

[bib15] Hasson U., Yang E., Vallines I., Heeger D.J., Rubin N. (2008). A hierarchy of temporal receptive windows in human cortex. J. Neurosci..

[bib16] Hoshi E., Tanji J. (2000). Integration of target and body-part information in the premotor cortex when planning action. Nature.

[bib17] Kurata K., Wise S.P. (1988). Premotor and supplementary motor cortex in rhesus monkeys: neuronal activity during externally- and internally-instructed motor tasks. Exp. Brain Res..

[bib18] Lerner Y., Honey C.J., Silbert L.J., Hasson U. (2011). Topographic mapping of a hierarchy of temporal receptive windows using a narrated story. J. Neurosci..

[bib19] Murray J.D., Bernacchia A., Freedman D.J., Romo R., Wallis J.D., Cai X., Padoa-Schioppa C., Pasternak T., Seo H., Lee D. (2014). A hierarchy of intrinsic timescales across primate cortex. Nat. Neurosci..

[bib20] Murray J.D., Bernacchia A., Roy N.A., Constantinidis C., Romo R., Wang X.-J. (2017). Stable population coding for working memory coexists with heterogeneous neural dynamics in prefrontal cortex. Proc. Natl. Acad. Sci. U S A.

[bib21] Mushiake H., Inase M., Tanji J. (1991). Neuronal activity in the primate premotor, supplementary, and precentral motor cortex during visually guided and internally determined sequential movements. J. Neurophysiol..

[bib22] Nakayama Y., Yamagata T., Tanji J., Hoshi E. (2008). Transformation of a virtual action plan into a motor plan in the premotor cortex. J. Neurosci..

[bib23] Nishida S., Tanaka T., Shibata T., Ikeda K., Aso T., Ogawa T. (2014). Discharge-rate persistence of baseline activity during fixation reflects maintenance of memory-period activity in the macaque posterior parietal cortex. Cereb. Cortex.

[bib24] Ogawa T., Komatsu H. (2010). Differential temporal storage capacity in the baseline activity of neurons in macaque frontal eye field and area V4. J. Neurophysiol..

[bib25] Tsujimoto S., Genovesio A., Wise S.P. (2011). Comparison of strategy signals in the dorsolateral and orbital prefrontal cortex. J. Neurosci..

[bib26] Tsujimoto S., Genovesio A., Wise S.P. (2012). Neuronal activity during a cued strategy task: comparison of dorsolateral, orbital, and polar prefrontal cortex. J. Neurosci..

[bib27] Tsujimoto S., Genovesio A. (2017). Firing variability of frontal pole neurons during a cued strategy task. J. Cogn. Neurosci..

[bib28] Yamashita Y., Tani J. (2008). Emergence of functional hierarchy in a multiple timescale neural network model: a humanoid robot experiment. PLoS Comput. Biol..

[bib29] Zylberberg J., Strowbridge B.W. (2017). Mechanisms of persistent activity in cortical circuits: possible neural substrates for working memory. Annu. Rev. Neurosci..

