# Supplementary material for: Neural Intrinsic Timescales in the Macaque Dorsal Premotor Cortex Predict the Strength of Spatial Response Coding
Source: iScience. 2018 Nov 22;10:203–10. doi: 10.1016/j.isci.2018.11.033 (PMC6287088; doi:10.1016/j.isci.2018.11.033)
Supplement: Document S1. Transparent Methods and Figure S1 [file mmc1.pdf]

ISCI, Volume *10*

Supplemental Information

Neural Intrinsic Timescales in the Macaque Dorsal Premotor Cortex Predict the Strength of Spatial Response Coding

Rossella Cirillo, Valeria Fascianelli, Lorenzo Ferrucci, and Aldo Genovesio

SUPPLEMENTAL INFORMATION

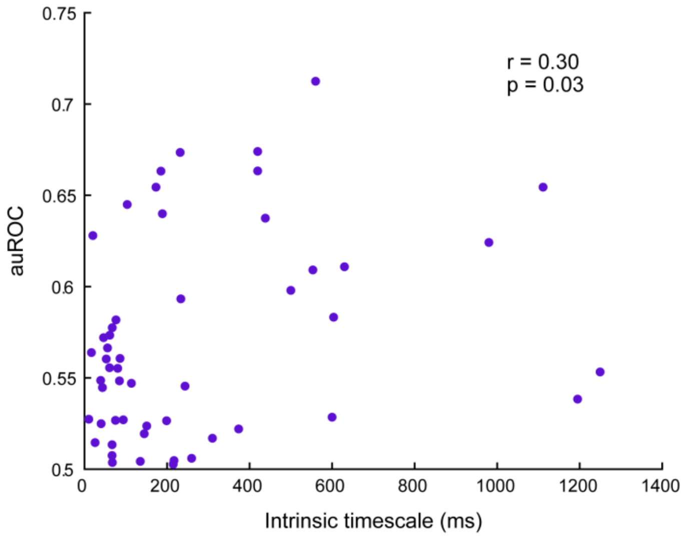

Figure S1. Scatter plot of the intrinsic timescale *vs.* auROC values. Related to Figure 5.
Scatter plot of the intrinsic timescales versus auROC values for all neurons (*n* = 52). Each dot represents a neuron. The Pearson correlation coefficient was computed (r=0.30; p=0.03).

TRANSPARENT METHODS

Animals. Animal care, housing and experimental procedures conformed to the European (Directive 210/63/EU) and Italian (DD.LL. 116/92 and 26/14) laws on the use of non-human primates in scientific research. The research protocol was approved by the Italian Health Ministry (Central Direction for the Veterinary Service). The housing conditions and experimental procedures were in accordance with the European law on humane care and use of laboratory animals. Two male rhesus monkeys (*Macaca mulatta*) served as subjects in this study, Monkey 1 (8 years old, 8 kg) and Monkey 2 (12 years old, 9.5 kg).

Behavioral task. We trained two male rhesus monkeys (*Macaca mulatta*) to perform a non-match-to-goal (NMTG) task (Cirillo et al., 2018). We used two versions of the task, that differed only in the peripheral stimuli. The duration of the task periods and the basic rule used to solve it were identical in both versions. The first version of the task, a spatial NMTG task (S-NMTG), was performed by Monkey 1 and Monkey 2. The latter monkey performed also an object NMTG task (O-NMTG). To obtain a comparable performance from the two monkeys, S-NMTG was used for Monkey 1 and O-NMTG for Monkey 2. Each monkey sat in a primate chair, head-restrained, facing a touch screen monitor (Microtouch, 19 inches, 800 X 600 pixel resolution) 20 cm away. In both versions of the task, each trial started when a red stimulus (a 7° diameter circle) appeared at the center of the screen. The monkeys had to touch the central stimulus within 2 s otherwise the trial was aborted and a new trial started. The monkeys had to hold the central stimulus for 500 or 800 ms (holding central stimulus). Consequently, in the S-NMTG task, two peripheral targets (see Fig. 1 left panel), represented by identical grey rectangles (7.1° X 7.7°), appeared in two of four possible screen positions: center left (23.5° left of center), bottom left (17.5° below and 23.5° left of center), center right (23.5° right of center) and bottom right (17.5° below and 23.5° right of center). In the O-NMTG task (Fig. 1), the peripheral stimuli were represented by four objects, differing in color and shape, that were paired and appeared one to the right and one to the left of the central stimulus (23.5° right/left of center). In both versions of the task, the appearance of the peripheral targets coincided with the beginning of a delay period of 800 or 1200 ms. During the delay period the monkeys had to continue touching the central stimulus until its disappearance. This represented a 'go' signal, which instructed the monkeys to select one of the two peripheral targets. After target selection, the monkeys had to hold the target for a fixed period of 400 or 600 ms. After each correct trial, the monkeys received water with juice as reward. An intertrial interval lasting from 1000 up to 1500 ms followed both correct and incorrect trials, during which the screen was totally black. On the next trial, the previously chosen target was presented again on the video screen with another target, randomly chosen from the three remaining targets. This could be either a new target or the target not chosen in the preceding trial. The task rule required to discard the previously selected target and select the alternative one. If the monkeys chose the same target that was selected in the previous trial, this represented an error trial and the animals did not receive the reward, and a correction trial followed. Correction trials consisted of the presentation of the same pair of targets presented in the immediately preceding and incorrectly performed trial. If an error occurred in a correction trial, another correction trial followed. The first trial choice of each session was always accepted as correct, with reward delivery for any chosen target.

The NMTG task as described by Cirillo et al. (2018) included also the interaction with a human agent: in some trials the monkey observed the human partner performing the task in his place. In the present work, we discarded all the trials performed by the human partner.

Surgical techniques. Before the training period, a head holding device was implanted in both monkeys. All surgical procedures were performed using aseptic conditions, under general anesthesia with isoflurane (Abbott Laboratories) through a constant flux of isoflurane/O2 mixture (1-3%, to effect). Antibiotics and analgesics were administered postoperatively. Before the recording started, a recording chronic Utah array of 10 X 10 electrodes was implanted stereotaxically.

The chronic array was implanted over the left dorsal premotor cortex (PMd) of both monkeys. Recording sites were localized relative to the arcuate and principal sulci both in Monkey 1 and Monkey 2 after opening the dura matter for the implant of the chronic array, and based on stereotaxic coordinates (see Cirillo et al., 2018 for details).

Neuronal data collection. To control stimuli presentation and reward delivery, and to detect touches on the screen and categorize the trials, we used a non-commercial software, CORTEX (http://www.nimh.nih.gov/labs-at-nimh/research-areas/clinics-and-labs/ln/shn/software-projects.shtml). We monitored the eye position through

the ViewPoint Eye Tracker system (Arrington Research, Scottsdale, USA). Neural activity of single units was recorded extracellularly with a 96-channel high-density microelectrodes chronic system (CerePort Utah Array; Blackrock Microsystems, LLC). The array (10x10 grid of 1.5 mm electrodes) was inserted during the surgery with a microelectrode array inserter (Blackrock) after opening the dura mater. The array was wired to a connector (16.5 mm height, 19 mm diameter base, 11 mm diameter body). Electrical signals were amplified and filtered, and single units were isolated online with a TDT system (Tucker-Davis Technologies, TDT, Alachua, USA). Neurons that were not well isolated, after the offline control (OpenSorter, TDT and Plexon Offline Sorter V3), were discarded. The same TDT system was used to record eye movements.

Data analysis

Neuronal analysis. All neurophysiological analyses were performed on the activity of neurons in correct trials. We analyzed the trials performed by the monkeys, discarding the human partner trials. We recorded 400 single neurons in PMd while the monkeys performed the NMTG task: 248 cells from Monkey 1 and 152 from Monkey 2. From the initial population of neurons, we selected a subpopulation of 328 cells – 210 from Monkey 1 and 118 from Monkey 2 – using a single-unit stability method to consider only units that were not the same across recording sessions (see Cirillo et al., 2018).

We selected trials with both holding central stimulus durations (500, 800 ms) and both delay durations (800, 1200 ms). We analyzed the neural activity during two task periods: the first 500 ms of the holding central stimulus period (considered as the “baseline period”) and the first 800 ms of the delay period (we refer to this time window as “delay period”).

Only neurons satisfying the following requirements were analyzed (Murray at al., 2014):

1. Recorded in at least 20 correct trials;
2. Each disjoint 50-ms bin during the baseline period with non-zero mean activity across trials. (1)

MatLab software (The MathWork, Inc., Natick, MA, USA) was used to perform all the analyses.

Autocorrelation structure and neural intrinsic timescale in the baseline period. The analysis of the autocorrelation of spike-count constituted the focus of our study. We assessed the autocorrelation structure as follows: we subdivided the first 500 ms of the baseline period into disjoint time bins of 50 ms. We then computed, for each neuron, the across trial autocorrelation between two time bins *i* and *j* (*i*, *j*, integer numbers) – placed at time lag of $|i\text{-}j| \times \Delta$ (Δ = 50 ms) – with the Pearson’s correlation coefficient, ρ (Murray et al., 2014):

$$\rho = \frac{Cov(N(i),N(j))}{\sqrt{Var(N(i)) \times Var(N(j))}} = \frac{<(N(i)-\overline{N}(i))(N(j)-\overline{N}(j))>}{\sqrt{Var(N(i)) \times Var(N(j))}} \qquad (2)$$

where, *N(i)* and *N(j)* are the spike-counts in the *i* and *j* time bins, respectively, and $\overline{N}(i)$ and $\overline{N}(j)$ are the mean spike-counts across trials for the same time bins *i* and *j*. The covariance (*Cov*) and variance (*Var*) were computed across trials for the *i* and *j* bins. All pair combinations of bins at various time lags were taken into account in order to calculate the coefficient ρ.

Subsequently, we computed the autocorrelation structure at the population level, averaging the coefficient ρ at each time lag across neurons. We then fit the autocorrelation values, as a function of time lag, to the population of neurons with an exponential function (Murray et al., 2014) defined as follows:

$$\rho(k\Delta) = A[\exp\left(-\frac{k\Delta}{\tau}\right) + B] \qquad (3)$$

where $k\Delta$ refers to the time lag between i and j bins ($k = |i - j|$, $k = 1, 2 \ldots 9$), ρ is the Pearson's correlation coefficient at time lag $k\Delta$, A is the amplitude, τ is the intrinsic timescale (decay constant), and B is the offset value that reflects the contribution of long timescales (much longer than our 500 ms time window) (Murray et al., 2014). From an analytic point of view, the decay constant τ is the time at which the autocorrelation is reduced to $1/e = 0.37$ (e is the Euler's number) times its initial value.

The same fit procedure was applied to single-neuron autocorrelation structure in order to assign the timescale τ obtained from Equation 3 to each cell. Neurons that did not satisfy both the criteria listed in (1) and the following criteria were excluded from further analyses:

1. intrinsic timescale $\tau > 0$;
2. amplitude $A > 0$. (4)

The first requirement is because a 0 ms or negative intrinsic timescale is meaningless. The second requirement imposes no reflection of the exponential function with respect the horizontal axis. Indeed, in case of a negative amplitude, the exponential function would increase with larger time lag and it does not depict the shape of the autocorrelation structure we are modeling – an autocorrelation structure exponentially decaying with increasing time lag.

Furthermore, we observed that neurons had a maximum value of their autocorrelation values in the range between 50 and 200 ms time lag after central stimulus onset. To accommodate this feature, the fitting procedure started at that time lag within the first 200 ms time lags (i.e., 50, 100, 150, or 200 ms) after which the autocorrelation value decreased.

From the population of neurons satisfying all the criteria listed in (1) and (4), we further selected those having an adjust R^2, associated to the fit, larger than 0.5. This value was a tradeoff between the willingness to keep the highest number of neurons and in parallel to guarantee a good fit result for each of them.

Multiple-linear regression on neural population. To test the relationship of the mean firing rate and the autocorrelation values, both computed in the 500 ms of the baseline period, with the strength of neuronal selectivity calculated in the delay period, we performed a multiple-linear regression analysis.

Neuronal selectivity represents the ability of a neuron to respond to a particular stimulus dimension. Preferred and anti-preferred conditions represent the dimensions for which a neuron shows the highest and the lowest tuning, respectively. To quantify the strength of the selectivity for the spatial response (right vs. left), we performed a receiver operating characteristic (ROC) analysis, which quantifies how strongly a neuron encodes a variable (Dayan and Abbott, 2005). We then computed the area under the ROC curve (auROC) that served as selectivity index. The auROC values ranged from 0 to 1, where 0 and 1 indicated the maximum selectivity for the opposing preferences. The normalized values of auROC were computed with respect to the preferred condition - that is, highest activity - and ranged from 0.5 (no selectivity) to 1 (maximum selectivity).

We performed a multiple-linear regression analysis on all the population of neurons satisfying the requirements listed in (1). For each neuron of the population we computed the firing rate averaged across trials in the 500 ms of holding central stimulus (baseline), the autocorrelation coefficient ρ (see Formula 2) computed in the 500 ms of baseline at 100, 200, and 300 ms time lags, and the normalized auROC computed in a 400 ms fixed time window from 400 to 800 ms after peripheral target onset for the spatial response (right or left; see Figure 1). We chose this time interval based on the results shown by Cirillo et al. (2018) in which we found a populations of neurons in PMd encoding the spatial response during the delay period. The independent variables were the mean firing rates and the autocorrelation values, and the dependent variable was the auROC. In the multiple-linear regression, the level of significance of each estimated parameter - to be significantly different from 0 - was set to 0.05.

Analysis of the relationship between the neural intrinsic timescale and the strength of neural selectivity. We assigned the intrinsic timescale τ to each neuron satisfying both the criteria listed in (1) and (4) and having an adjust R^2 from the exponential fit larger than 0.5. These neurons represented a neural subsample on which we investigated whether a longer intrinsic timescale computed in a baseline period was predictive of a stronger neural selectivity in a following task period (delay period).

From this sample, we further removed those neurons having an intrinsic timescale classified as outlier according to the distribution of the intrinsic timescales.

To identify an outlier, we used the interquartile range (IQR) method. The IQR is the range between the first and the third quartiles. We labeled any timescale value that fell outside of either 1.5 times the IQR below the first – or 1.5 times the IQR above the third – quartile as outlier. We decided to remove outliers after a visual inspection of the intrinsic timescale distribution. We observed some neurons with very long intrinsic timescale (> 1500 ms) representing a linear decay of the autocorrelation structure in the time window we examined (500 ms baseline) rather than an exponential decay as we modeled.

First, for each of the remaining neurons, we calculated the normalized auROC for the spatial response (right - left) in a 400 ms fixed time window from 400 to 800 ms after peripheral target onset (see Figure 1) (Cirillo et al., 2018).

Subsequently, we tested the relationship of intrinsic timescale and mean firing rate in the baseline period with the auROC. The intrinsic timescale and the mean firing rate were regressed onto the auROC of each neuron.

To better illustrate the relation between the intrinsic timescale and the strength of the neural encoding along time, we sorted the neurons according to increasing timescales and split them up in 2 groups by the median value: long and short timescale populations, defined as neurons with a timescale above and below the median value, respectively (Cavanagh et al., 2016). To examine the time course of neuronal selectivity, we calculated the normalized auROC values for the long and short timescale populations using a sliding window of 400 ms, increasing in steps of 20 ms aligned on peripheral targets onset (Figure 1). Subsequently, the normalized auROC values were averaged across neurons within each timescale population.

Supplemental References

Cavanagh, S.E., Wallis, J.D., Kennerley, S.W., and Hunt, L.T. (2016). Autocorrelation structure at rest predicts value correlates of single neurons during reward-guided choice. Elife *5*, 1–17.

Cirillo, R., Ferrucci, L., Marcos, E., Ferraina, S., and Genovesio, A. (2018). Coding of Self and Other's Future Choices in Dorsal Premotor Cortex during Social Interaction. Cell Rep. *24*, 1679–1686.

Dayan, P., and Abbott, L.F. (2005). Theoretical Neuroscience: Computational and Mathematical Modeling of Neural Systems. MIT Press Ltd.

Murray, J.D., Bernacchia, A., Freedman, D.J., Romo, R., Wallis, J.D., Cai, X., Padoa-Schioppa, C., Pasternak, T., Seo, H., Lee, D., et al. (2014). A hierarchy of intrinsic timescales across primate cortex. Nat. Neurosci. *17*, 1661–1663.
